# From shock to resilience in GBV and SRH services: the role of research

**DOI:** 10.1136/bmjgh-2025-021152

**Published:** 2026-01-08

**Authors:** Jonathan Kaunda Mwansa, Miriam Hartmann, Mariano Salazar, Monica Chibesakunda, Natalie Vlahakis, Anna Mia Ekström, Mwenya Mubanga

**Affiliations:** 1 University of Lusaka, Lusaka, Zambia; 2 USAID Stop GBV Now Project, Lusaka, Zambia; 3 Department of Global Public Health, Karolinska Institutet, Stockholm, Sweden; 4 Women’s Global Health Imperative, RTI International Berkeley Office, Berkeley, California, USA; 5 Centre for Infectious Disease Research in Zambia, Lusaka, Lusaka Province, Zambia; 6 Department of Medical Epidemiology and Biostatistics, Karolinska Institutet, Stockholm, Sweden

**Keywords:** Gender-Based Violence, Global Health, Health systems, Africa

**Summary box**

- Abrupt US Agency for International Development funding cuts led to the collapse of Zambia’s Stamping Out and Preventing Gender-Based Violence programme, with police data showing rising reports of sexual violence.
- External shocks intensify gender-based violence and disrupt sexual and reproductive health services, as seen during COVID-19, with long-term adverse outcomes.
- Research must move beyond documenting collapse to identifying resilience mechanisms, for example, who is most affected; how integration can be sustained; and how systems absorb, adapt and transform under volatility.
- Systems thinking and the Dynamic Sustainability Framework offer tools for strengthening absorptive, adaptive and transformative capacities that sustain multisectoral integration under changing conditions.
- Future partnerships should prioritise locally led, coproduced and implementation-focused research that embeds learning within service delivery and links evidence to policy and community accountability.

In January 2025, Zambia’s Stamping Out and Preventing Gender-Based Violence (STOP GBV) programme, one of the country’s most comprehensive multisectoral initiatives, abruptly halted after US Agency for International Development (USAID) suspended funding due to changing political landscapes.^1^ Within months, shelters closed, access to HIV prophylaxis diminished and thousands of survivors were left without support. This funding shock disrupted gender-based violence (GBV) and sexual and reproductive health (SRH) services and exposed critical gaps in how public health research anticipates and responds to external shocks.

The STOP GBV programme’s scale and reach made it one of Zambia’s most comprehensive and promising multisectoral health and social interventions. Since its launch in 2005, STOP GBV had expanded to 56 of Zambia’s 116 districts. Through a One-Stop Centre model, it integrated trauma counselling, legal and paralegal support, HIV testing and prevention, occupational therapy and reintegration services. This trauma-informed, person-centred approach linked survivors with health services, the police, judiciary and community networks. By 2024, the programme supported more than 82 000 survivors, trained over 5000 frontline workers, established a shared data platform and ran 41 centres nationwide with an annual budget of US$4 million.^2^

The sudden closure of STOP GBV appears to have alarming consequences in a setting where women and girls face persistently high rates of lifetime violence. In normal times, one out of five children experience sexual abuse, and nearly half (47%) of ever married women aged 15–49 have experienced intimate partner violence.^3^ Heightening this context, quarterly data from the Zambia Police Service in regions previously supported by USAID show a 15.2% increase in reported GBV cases between January and March 2025 compared with the same period in the previous year. Most alarming, 2474 of these cases involved children under 15, 73% of them girls.^4^ With key services no longer available, many of these survivors are now left without HIV postexposure prophylaxis, forensic examinations, psychosocial and paralegal support or proper referral pathways.^5^

The shutdown echoes earlier crises, which highlight the consequences on GBV, on broader SRH, and points to the role of worsened systematic drivers. During COVID-19, Zambia, like many sub-Saharan African countries, experienced reported surges in GBV as services were disrupted.^5^ In South Africa, longitudinal research showed that violence was a direct result of lockdowns and intensified by systemic shocks such as rising unemployment, food insecurity and worsening mental health.^6^ In Zambia, service disruptions meant more women missed antenatal care, increasing the risk of negative birth outcomes,^7^ while across multiple low- and middle-income countries, adolescent girls saw a rise in unintended pregnancies due to school closures and lack of SRH access.^8^ These examples illustrate how external shocks expose vulnerabilities across interconnected economic, health and education systems, with disproportionate impacts on women and girls at different life stages.

The next disruption, whether political, climate or health related, is inevitable. Yet, documenting collapse is not enough. Funding shocks such as the closure of Zambia’s STOP GBV programme reveal that resilience depends on resources and on how systems learn, adapt and protect those most at risk. Resilience is a process that requires both a systems thinking perspective, recognising how disruptions in one part of the health system may impact others,^9^ and a theoretical framework, guiding it such as the Dynamic Sustainability Framework (DSF).^10^ The DSF highlights that changes over time in the characteristics of a given system (eg, financial resources) shape how interventions, such as Zambia’s STOP GBV programme, are implemented, and embeds the idea that continuous learning, adaption and improvement are necessary for sustainable system integration.^10^

Building health system resilience in Zambia and elsewhere should thus focus on strengthening the core capacities underpinning it, including:

1. Maintaining essential functions and protecting populations during shocks (absorptive capacity).
2. Adjusting resources, relationships and processes to changing conditions (adaptive capacity).
3. Reorganising health system structures when existing arrangements no longer suffice (transformative capacity).^11^

In resilience research, these capacities are understood as interdependent and dynamic rather than distinct silos. They describe how systems respond to stress over time: absorptive capacity enables continuity during shocks; adaptive capacity allows adjustment and learning as conditions change; and transformative capacity supports the reorganisation of structures when existing arrangements are no longer viable. The DSF provides a way to operationalise these ideas by emphasising ongoing learning and alignment between interventions and the systems in which they are embedded. In practice, these capacities can be strengthened through three complementary domains of resilience: financial, knowledge and political, each of which represents a point where research and policy can sustain integration under volatility.

Applying these capacities to the current Zambian situation means building financial resilience (*absorptive capacity*) by ensuring that services for GBV survivors are funded by different complementary mechanisms to avoid the collapse of services when one mechanism fails. For example, research on South Africa’s Thuthuzela Care Centres has illustrated how multidepartmental funding supports service continuity, providing evidence that can guide similar system-embedded financing in other contexts.^12^

In addition, the *adaptive capacity* of the Zambian health system should be improved by increasing its research and implementation capacities and embedding them within service delivery, testing rapid feedback mechanisms, coproducing data dashboards and integrating operational data across sectors so that evidence informs implementation in real time leading to knowledge resilience. For example, during COVID-19, locally led studies in South Africa and Zambia documented disruptions to GBV and SRH services, revealing system fragilities and inequities in access.^5^,^7^ In South Africa, emerging data during COVID-19 informed public debate and advocacy around the National Strategic Plan on Gender-Based Violence and Femicide, yet institutional uptake of evidence was fragmented and constrained by gaps in performance data and coordination, highlighting their importance.^13^

Finally, the *transformative capacity* of the Zambian health system can be improved by strengthening the existent mechanisms for engaging local actors and stakeholders in the cocreation, implementation and evaluation of GBV prevention programmes contributing to political resilience. Public health research contributes by codeveloping and evaluating mechanisms for community engagement, policy, uptake and transparency. For instance, civic data platforms such as OpenUp^14^ in South Africa demonstrate how researchers, citizens and policymakers can jointly design and monitor service indicators, institutionalising feedback loops that sustain political will and systemic responsiveness.

For the STOP GBV programme in Zambia, the opportunity to build resilience into the system passed with its termination due to the funding cuts. As the Government works to rebuild these vital services, public health research must move beyond describing shocks and disruptions. Through work that maps system financing, embeds learning within service delivery and codevelops accountability mechanisms, researchers can help integrate financial, knowledge and political resilience into GBV and SRH systems. Ultimately, locally led, implementation-focused and embedded research that generates actionable evidence for sustaining essential services under conditions of uncertainty is critical to protecting progress in GBV and SRH.

## Data Availability

There are no data in this work.
